# Evolutionary flexibility of protein complexes

**DOI:** 10.1186/1471-2148-9-155

**Published:** 2009-07-07

**Authors:** Michael F Seidl, Jörg Schultz

**Affiliations:** 1 Department of Bioinformatics, Biozentrum, University Würzburg, Am Hubland, 97074 Würzburg, Germany; 2 Theoretical Biology and Bioinformatics Group, Department of Biology, Faculty of Science, Utrecht University, Padualaan 8, 3584CE Utrecht, The Netherlands

## Abstract

**Background:**

Proteins play a key role in cellular life. They do not act alone but are organised in complexes. Throughout the life of a cell, complexes are dynamic in their composition due to attachments and shared components. Experimental and computational evidence indicate that consecutive addition and secondary losses of components played a major role in the evolution of some complexes, mostly without affecting the core function. Here, we analysed in a large scale approach whether this flexibility in evolution is only limited to a distinct number of complexes or represents a more general trend.

**Results:**

Focussing on human protein complexes, we based our analysis on a manually curated dataset from HPRD. In total, 1,060 complexes with 6,136 proteins from 2,187 unique genes were considered. We computed interologs in 25 different species and predicted the composition of complexes. Over the analysed species, the composition of most complexes was highly flexible and only 25% of all genes were never lost. Even if one component was lost at a particular point in time, the fraction of observed second, independent losses of additional components was high (75% of all complexes affected). Still, loss of whole complexes happened rarely. This biological signal deviated significantly from random models. We exemplified this trend on the anaphase promoting complex (APC) where a core is highly conserved throughout all metazoans, but flexibility in certain components is observable.

**Conclusion:**

Consecutive additions and losses of distinct units is a fundamental process in the evolution of protein complexes. These evolutionary events affecting genes coding for units in human protein complexes showed a significantly different phylogenetic pattern compared to randomly selected genes. Determination of taxon specific attachments or losses might be linked to specific cellular or morphological features. Thus, protein complexes contain not only structural and functional, but also evolutionary cores.

## Background

Proteins are, next to RNA, the fundamental unit of biological activity. But, they do not act alone. Many biological and cellular processes require a precise organisation of proteins in time and space [1]. These multi protein complexes, also called molecular- or protein-machines, are among the fundamental entities of molecular organisation [1,2]. Recent high throughput studies identified and analysed the components of protein interaction networks and how they are organised to functional units [1,3-5]. On a higher level, multi-protein complexes are embedded in a network linking cellular processes [6]. Here, the complexes are connected by shared components, e.g. proteins present in more than one complex. Most of these shared components are associated peripherally and are not integral members of the complexes suggesting a role in the regulation of molecular-machines [6]. Complementary to this network view, protein complexes can be partitioned in a core which is modulated by different attachments. By adding different attachments, isoforms of a complex are built, possibly with slightly different functions. Some of these attachments, which can consist of multiple proteins itself, can be connected to different core complexes. These mobile regulatory units are often called modules [1]. The combination of core functional units with variably attached modules increases the number of different complexes and thereby the complexity of the cell. This complexity, comprising both the functional and structural entities of protein complexes, raises the question how the interplay of core complexes with variable attachments evolved. As a first step in this direction, it has been shown that yeast complexes enriched with gene products having an ortholog in human preferentially interact with other gene products that also have a human ortholog [3]. Comparing the constitution of cores and modules in other species revealed that they are unlikely to be present partially [1]. This could be interpreted as an 'ortholog proteome' that resembles the backbone necessary to facilitate fundamental functions of an eukaryotic cell [7].

Complementary to these large scale analyses, an in-depth study of the SMN complex which is involved in splicing revealed a high degree of evolutionary flexibility of its components [8]. The studied complex is responsible for mediating assembling of the UsnRNPs (uridine rich small nuclear ribonucleoproteins). In humans, it consists of eight components, namely SMN and the Gemins 2–8. This complexity arose via addition of distinct entities to the ancestral core of SMN and Gemin 2 which can already be found in protists. Contrary to this trend, diptera have lost three of the components but still contain a functional SMN complex. Similar losses were found in further organism, indicating evolutionary dynamics of the complex.

Here, we addressed the question whether evolutionary flexibility is limited to a distinct number of machines or represents a general feature of the evolution of protein complexes.

## Results and discussion

### A parsimony based approach for inferring the evolutionary history of protein complexes

**Figure 1:**
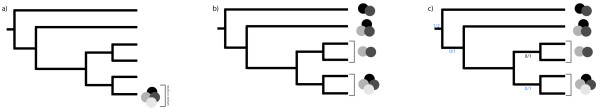
**Identification of 'ortholog' complexes and their evolutionary history**. Example explaining the identification of 'ortholog' complexes and the maximum parsimony approach to infer the evolutionary history according to a phylogenetic tree. A hypothetical complex consisting of four components is derived from HPRD (a). Computing the ortholog genes using InParanoid and deriving the constitution of the complex in all species of interest (b). Using a maximum parsimony approach to infer the evolutionary history, gene emergence and loss events, of every component of the complex. The numbers in blue indicate complex or gene emergence, the black numbers loss events (c).

We focussed our analysis on human protein complexes annotated in the human protein reference database (HPRD), as this database is manually curated and, accordingly, of high quality [9]. At the time of the analysis, the HPRD dataset contained 2,197 distinct genes which were found in 1,060 protein complexes. As a first step, we identified orthologs of these genes in the genomes of a selected subset of species (see Fig. 1a–c for a hypothetical example of the applied approach). To provide a wide spectrum, we chose 25 annotated eukaryotic species including 17 metazoan, six fungi, one choanoflagellate and one amoebozoa as an outgroup (see Tab. 1). Using literature data, a phylogenetic tree for these species was reconstructed (see Methods). For ortholog detection InParanoid [10] combined with an iterative searching approach was implemented (see Methods for details). Using the concept of interolog mapping [11,12] allowed the prediction of the constitution of 'orthologous' complexes in each species (see Fig. 1b). This prediction will vary from the 'real' complex, as we did not consider gene duplications. A duplication in the other (non human) species should not influence the results, as one of the copies is expected to stay as a member of the protein complex. If the duplication is human specific, two scenarios have to be distinguished. In the first, both human genes are components of different protein complexes. In this case, their ancestor was probably a member of both complexes [13]. In the second scenario, only one of the duplicated proteins is a member of a complex. In cases where this functionality evolved after the speciation, a false positive will be seen. Thus, gene duplications will only slightly influence the prediction of the 'ortholog' complexes. Based on the presence and absence pattern of complexes and the forming components we inferred the evolutionary history using on a parsimony based approach (see Methods and Fig. 1c for more information).

**Table 1:** Table of the examined species, the source and the version.

Name	Version	Release date	Source	Reference
*Anopheles gambiae*	AgamP3	Feb. 2006	Ensembl	[35]
*Apis mellifera*	v2.0	unknown	Beebase	[36]
*Aspergillus niger*	v1.0	Nov. 2005	JGI	-
*Branchiostoma floridae*	v1.0	Mar. 2006	JGI	[37]
*Caenorhabditis elegans*	WS180	Sep. 2007	Ensembl	[38]
*Ciona intestinalis*	JGI2	Mar. 2005	Ensembl	[39]
*Danio rerio*	ZFISH7	Jul. 2006	Ensembl	[40]
*Daphnia pulex*	v1.0	Sep. 2006	JGI	-
*Dictyostelium discoideum*	unknown	Jan. 2008	Dictybase	[41]
*Drosophila melanogaster*	BDGP4-3	Jan. 2006	Ensembl	[42]
*Encephalitozoon cuniculi*	unknown	Jan. 08	NCBI	[18]
*Homo sapiens*	NCBI36	Nov. 2006	Ensembl	[14,15]
*Laccaria bicolor*	v1.0	Mar. 2005	JGI	[43]
*Monosiga brevicollis*	v1.0	Jul. 2006	JGI	[44]
*Mus musculus*	NCBIM37	Apr. 2007	Ensembl	[45]
*Nematostella vectensis*	v1.0	2006	JGI	[46]
*Oryzias latipes*	MEDAKA1	Oct. 2005	Ensembl	[47]
*Phycomyces blakesleeanus*	v1.0	Sep. 2006	JGI	-
*Rattus norvegicus*	RGSC3-4	Nov. 2004	Ensembl	[48]
*Saccharomyces cerevisiae*	SGD1	Dec. 2006	Ensembl	[49]
*Schizosaccharomyces pombe*	v19.0	unknown	Sanger	[50]
*Takifugu rubripes*	FUGU4	Jun. 2005	Ensembl	[51]
*Tetraodon nigroviridis*	TETRAODON7	Apr. 2003	Ensembl	[52]
*Trichoplax adhaerens*	v1.0	Jul. 2006	JGI	[53]
*Xenopus tropicalis*	JGI4-1	Aug. 2005	Ensembl	-

Names of the examined species in alphabetical order, the source (Ensembl, JGI, species related databases), the version, the release date and the reference if available.

### Emergence of protein complexes and their components

**Figure 2:**
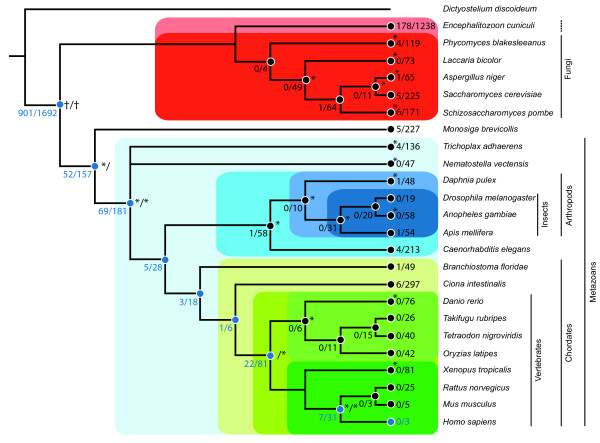
**Phylogenetic Tree with gene and complex emergence and losses**. The pattern of gene and complex emergence and the secondary losses of components of whole complexes is displayed along the tree according to the absence and presence pattern of the ortholog genes in terminal species or in subsets of species concluding the loss in the last common ancestor of all subsequent species. The numbers of gene and complex emergence is indicated in blue (complex emergence/gene emergence). The number of secondary losses are shown in black per affected node. It was discriminated between whole complex losses and gene losses (complex losses/gene losses). The significance of emergence (discriminated between complex and gene emergence) and loss (only gene) events compared to the random model are indicated with '*'. As we restricted our analysis to fungi and metazoans, evolutionary events which have been mapped to the base of the tree ('†') could have evolved at any time before the split.

As a first step, the emergence of each gene coding for a component was reconstructed according to the species tree (Fig. 2, blue numbers). For 77% of the genes orthologs were found in at least one fungus, indicating that their origin lay before the split of fungi and metazoans. Branches with a substantial addition of orthologs were the base of choanoflagellates-metazoans (157) and from there to the metazoan lineage (181). Based on the species sampling, these 'inventions' could also represent fungi specific gene losses. It has been suggested that the observable complexity of organisms is not mainly reflected by the gene number [14,15] but, among many other factors, by the number of protein interactions and the resulting interaction networks [6]. Indeed, the estimated size of different interactomes, in which protein complexes are embedded [6], is correlated with the biological complexity [16]. Thus, the emergence of genes co-localises with the increase in morphological complexity and the evolution of certain traits, like for the vertebrates (81) and mammals (31).

In a second step, we focused on the more complex centric view and analysed the emergence of whole complexes. We applied three alternative definitions specifying the emergence of a complex. (i) The point where at the first time two or more components of the complex were found (subsequently added or present at once), according to a definition that at least two components are necessary to constitute a complex [17]. (ii) The point of occurrence of the largest set of components at one time. (iii) The point of occurrence of all HPRD annotated components. Obviously, these definitions are oversimplifications as the minimal number of components necessary to constitute a functional complex could be different for every complex. Still, with our definitions we provided an upper and lower boundary to estimate complex emergence. With the most general definition, most of the complexes were already present in the last common ancestor of human and fungi (approximately 85%), with an increase at the base of the choanoflagellates-metazoans lineage, the metazoans, vertebrates and mammalians, respectively (Fig. 2). Comparable results were found with the second definition. Even with the most conservative definition a high number of complexes were observable at the last common ancestor of human and fungi or before (approximately 42%) and huge accretions at the base of the choanoflagellates-metazoans lineage (not considering fungi specific gene losses) and the metazoans. Overall, nearly 82% of all complexes had already emerged at that point. To test whether our results reflect an evolutionary signal and not just random fluctuations in complex composition we compared them to a random model. We chose a random subset of human genes identical in size to the original dataset and calculated the emergence of genes and complexes. This was repeated 10,000 times and compared to the biological signal. For most of the nodes (highlighted with a '*' in Fig. 2), the number of gene and complex emergence events differed significantly between the biological signal and the random model (all p-values smaller than an alpha (0.05) corrected for multiple testing, see Methods). In all significant nodes, fewer genes evolved than expected from the random model. Thus, a gene coding for a protein of a human complex tends to be older than the average gene.

The initial emergence of a complex is followed by a sequential addition of further components which might be linked to cellular or morphological features. Moreover, most components of protein complexes emerged early in the species tree and tend to be older than randomly chosen human genes.

### Secondary loss

Having calculated the point of emergence for each component of a human protein complex, we were now able to address the question of secondary losses of genes and whole complexes. For each gene present in a human protein complex, we predicted species missing its ortholog and, to identify the likely branch of gene loss, mapped gene losses to the last common ancestor. To test the significance of the observed pattern, we compared our results to a random model which took into account the observed bias of emergence events. In all significant cases (with *Aspergillus niger*, *Phycomyces blakesleeanus *and *Anopheles gambiae *as exceptions) fewer losses were observed than expected from the random model. Nevertheless, a high number of losses occurred along the tree (Fig. 2, black numbers). Interestingly, *Encephalitozoon cuniculi *has lost approximately 73.2% of the genes present in the last common ancestor of fungi and metazoan/choanoflagellates lineage. This might be the result of the intracellular parasitic nature with a reduced gene set, complete losses of biochemical pathways and a reduced protein-protein interaction network [18]. Comparable, but not equally large gene losses were observed in *Saccharomyces cerevisiae*, *Monosiga brevicollis*, *Trichoplax adhaerens*, *Caenorhabditis elegans *and *Ciona intestinalis*. A general trend for the loss of genes was already described for fungi, insects and *C. elegans *[19-21]. When looking only at genes with orthologs in human protein complexes we recall this trend for fungi and *C. elegans*. In contrast, we did not find any outstanding number of losses in insects in general or diptera in particular. The high number of losses found in *C. intestinalis *might be caused by errors in gene prediction. In the analysis of the SMN complex orthologs for *C. intestinalis *were not identified on the proteomic level due to annotation problems, but in a search against the whole genome shotgun sequences [8]. This example highlights the dependency of this analysis on the quality of the available genome data. Here, we focussed on proteins with a function in a protein complex which evolve comparably slow [22]. As most gene annotation pipelines utilize homology prediction, the rate of false positives will be lower than for randomly chosen proteins.

In total, only 25% of the genes found in human protein complexes were present in all species subsequent to the initial emergence. Of this 522 genes, 302 (approximately 58%) have already emerged before the fungi/metazoan split. The fraction of at least one secondary loss in the HPRD dataset of 2,197 human genes was 76.2%. This highlights the evolutionary flexibility of genes coding for components which are part of protein complexes. 913 genes were affected by more than one loss event, which is approximately 55% of all the genes affected by secondary losses. Thus, genes which are affected by a loss once, are more likely to be affected by additional further losses.

Nearly 44% of all 2,197 analysed genes were present in more than one complex and 36 of them were found in more than 10 different complexes. Of the nine genes that are shared between more than 15 complexes those with the highest occurrence were never lost, especially Integrin beta-1 precursor [Ensembl:ENSG00000150093] which is present in 54 complexes. The mean number of losses in genes that are present in more than 10 complexes was 1.25 (range 0–5), the mean number of losses found in only a single complex was 1.65 (range 0–13). Genes coding for proteins that are present in multiple complexes and therefore form a high number of interactions tend to evolve more slowly and seem to be more conserved than genes coding for proteins with few interactions, however the magnitude of difference was not dramatic [19,23]. Our analysis corroborates these observations.

Contrasting a high variability of the components of protein complexes, we rarely observed a loss of a whole complex. An exception was again *E. cuniculi*, which had lost many complexes completely. Thus, the loss of certain parts of already established complexes seems to be tolerable for the fitness of the organism. Overall, only 32 complexes annotated in HPRD (excluded complexes with the size of one) did not suffer from any secondary loss (3%) and 96.13% had at least one secondary loss of any component present (1,019). 75% of the complexes had at least two losses, indicating that functional modules or single components of different subunits were lost. Still, the core functionality of the complex has to be conserved, either as the result of the remained functionality or by the recruitment of non-ortholog, but functional equivalent, gene products. When predicting the composition of human complexes in other species, our analysis suggest that the composition is evolutionary highly flexible. However, the absence of whole complexes was rarely observed, indicating that either the remaining component are sufficient or additional, species specific, components are recruited to preserve the main function of the complex in the given context. In contrast, the partial loss or presence of ortholog components in different species in either core or modules has not been reported for yeast [1]. This difference might be the result of the heterogeneity of the HPRD datasets, comprising core, modules and attachments or the fact that the protein interaction network of human, compared to yeast, is larger, generating more hypothetical possibilities of flexibility.

### Evolutionary dynamics of the APC Complex

As a case study, we analysed the anaphase-promoting complex (APC), also called cyclosome, in detail. The APC plays a key role in the degradation of cyclines and other factors of cell cycle regulation mediated by the attachment of multiple ubiquitine chains to a lysine residue in the target protein (for a review on ubiquitination see [24]). The human cyclosome is a large, 1.5 MDa complex consisting of 11 core components (annotated in HPRD as 'COM_144'; one additional component, Apc13 is not described in HPRD) and two additional transient attachments (also not found in HPRD) required to bridge the interaction with the substrate [25] and activate the APC [26]. Two components, Apc2 and Apc11, built the catalytic core of the complex [25] and both are conserved throughout most eukaryotes and essential in the examined species [27,28]. The whole complex can be divided in four different sub-complexes, composed of the structural part (Apc1/Apc4/Apc5), the catalytic arm (Apc2/Apc11/Apc10), a tetratricopeptide repeat (TPR) arm (Apc8/Apc6/Apc3/Apc7/Cdc26/Apc13 [Ensembl:ENSG00000129055]) involved in adaptor binding and the attachments bridging the interaction to substrate (Cdc20/Cdh1; [Ensembl:ENSG00000117399]/[Ensembl:ENSG00000105325]).

We predicted the composition of the APC complex in 24 species using the described InParanoid procedure. For species where a loss was inferred we manually checked the absence of the particular gene product by using a reciprocal best hit approach against the NCBI non redundant database (nrdb).

**Figure 3:**
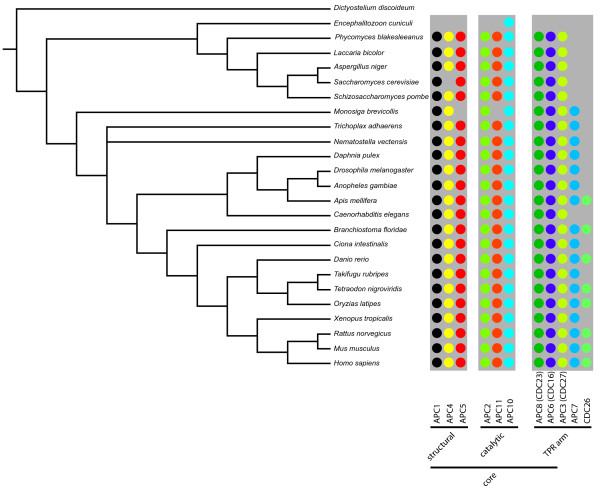
**The APC complex**. Graphical representation of the presence-absence pattern of single components of the APC complex, grouped by the sub-complexes (the composition of the sub-complexes have been derived from the literature [25] and is not reflected in HPRD). The structural, the catalytic and the TPR arm create the core complex. Presence of a component is indicated by a circle, the spectrum of examined species by the grey underlying bar (*D. discoideum *as outgroup was not considered).

The structural part of the complex was already present in the last common ancestor of human and fungi (Fig. 3, additional file 1 for the corresponding gene identifier). Apc1 was ubiquitous found in all species except *E. cuniculi*. The ortholog in *Danio rerio *was identified by a manual search against nrdb. Apc4 was lost in *E. cuniculi *and seems to be lost in *S. cerevisiae*. Experiments revealed a protein functionally corresponding to Apc4 in *S. cerevisiae*, but it was highly divergent and showed only a weak similarity to the human and the *Schizosaccharomyces pombe *Apc4 [27,28]. *E. cuniculi *and *M. brevicollis *have furthermore lost Apc5. The ortholog of Apc5 in *C. elegans *was not predicted by InParanoid, however could be inferred by a search against nrdb.

The components of the catalytic arm of the multi-protein enzyme were also present in the last common ancestor of fungi and human. Apc10, promoting substrate binding [25], was the most conserved subunit found in every examined species. Apc2 and Apc11, both part of the catalytic core, were identified throughout our species selection, except for *E. cuniculi *and in the case of Apc11 in *M. brevicollis*. The orthologs of Apc11 in *Xenopus tropicalis*, *Drosophila melanogaster *and *C. intestinalis *were identified by a manual search against nrdb.

The TPR arm components were also present in the last common ancestor of fungi and humans. Apc3, Apc6 and Apc8 were found in all analysed metazoan genomes and are even conserved throughout most fungi [25], highlighting the importance of the subunits to associate the attachments to the APC. Apc7, another component of the TPR arm sub-complex, has been described as vertebrate specific. Recent studies [29] indicated a genuine ortholog in *D. melanogaster*. We identified further orthologs in all metazoan and in *M. brevicollis *with the only exception of *C. elegans*. Additional orthologs were identified in plants and *Dictyostelium discoideum*. Thus, fungi seem to have lost this gene. The Cdc26 subunit, a small protein of 86 amino acids, was only identified in chordates and arthropods. Functional equivalents were described in *S. cerevisiae *(also named Cdc26) and *S. pombe *(named Hcn1) [30]. A manual PSI-BLAST [31] search with the *S. cerevisiae *Cdc26 protein and the *S. pombe *Hcn1, respectively, did not report any sequence similarity to other proteins in our dataset.

The APC complex demonstrate that both high evolutionary flexibility and conservation of entities in human complexes could be observed. Moreover, we show examples that the loss of a gene can be compensated by the displacement with a non-homologous gene product to sustain the functionality of the complex.

## Conclusion

How do protein complexes evolve? Do they emerge with all components at a specific branch in the phylogenetic tree or is it a more gradual process over longer time scale? Looking from human complexes back into phylogenetic history, we found that both is true. In most cases the emergence of some members of the complex is followed by the addition of further components. Still most components of protein complexes tend to be older than randomly chosen genes. Although the components show fewer losses than observed in a random model we also revealed frequent secondary losses of genes involved in a specific complex. Are these losses of genes with a possibly important function in the human complex real? A critical point in the analysis is the sequence based ortholog detection. If proteins evolve too fast, homologs might not be identified but still be present leading to false negatives and thereby to increased loss rates. An analysis of the BLAST algorithm underlying InParanoid showed that BLAST consistently identified homologs even over larger phylogenetic distances than used here [32]. We further improved sensitivity by using InParanoid, one of the best programs for ortholog detection [33] and applying iterative pairwise comparisons. Finally, the analysis focussed on proteins with a function in a complex which evolve slower than randomly selected proteins. We therefore expect only a small influence by false negative orthologs. We identified secondary gene losses on the sequence level, without the possibility to infer the function of the resulting complexes in the examined species. The SMN complex demonstrated that even with a reduced set of genes a complex can be still functional. Moreover, as seen in the APC complex, the loss of a gene can be compensated by the displacement with a non-homologous gene product. In many cases these enzymes have evolved by shifting the substrate specificity of a related but distinct enzyme [34].

Despite these limitation, our results indicate that losses can happen even for genes which are tightly bound into an interaction network like a protein complex. Together with the gradual emergence this has several consequences. First, one can identify an evolutionary core of a protein complex complementary to structural or functional cores. Second, taxon specific attachments or losses of complexes might be linked to specific cellular or morphological features. Third, the identification of the 'smallest' version of a complex might enable an easier experimental characterisation.

## Methods

### Genomic Data

Genomes used in this study as well as their source and version are given in Tab. 1[14,15,18,35-53].

### Species Tree

The phylogenetic tree used to guide the analysis and the ortholog identification was based on literature data. The position of *D. discoideum *as the outgroup to all other sampled species has been shown in [41] where a phylogeny based on ortholog clusters between different species had been calculated. The relationship of the fungi was derived from [54] where a concatenated six gene marker was used to infer the positions of the species. The position of the microsporidia (e.g. *E. cuniculi*) within the fungi is currently under debate, due to accelerated rate of sequence evolution. Early results suggested that microsporidia are among the earliest diverging protist lineages within the eukaryotes [55], however this seems to be an artefact of 'long branch attraction (LBA)' [56,57]. Recent phylogenetic [54,58,59] and molecular results [60-62] have implied that microsporidia are in fact atypical fungi [63] (Fig. 2 – red/light-red box). For the choanoflagellate *M. brevicollis *the position on the basis as the closest known relative to the metazoan clade was extracted from [44]. The basic relationship within the metazoan was found in [64](Fig. 2 – light-blue box). The nematod *C. elegans *was placed as a sister group to the arthropods, according to the ecdysozoa hypothesis (Fig. 2 – blue box). An analysis based on the coelomata hypothesis did not lead to substantially different results (supplemental material, additional file 2). The precise order in the arthropods was gathered from the honey bee genome publication [36], for the fishes from a phylogenomics approach focusing on the Hox gene cluster [65]. The position of the lancelets and the urochordates to the vertebrates was chosen based on recent molecular data, suggesting that the urochordates, and not the lancelets [66], are the closest relatives to vertebrates [67]. As the exact order of divergence of the placozoan and cnidaria has not been determined beyond doubts [68], it was represented as a trifurcation.

### Ortholog detection

For the analysis of the ortholog relationships we used InParanoid [10] in version 2.0, with standard parameters and an outgroup. The outgroup was chosen as the closest sister taxon of the compared species. The underlying BLAST search was performed with the usage of the '-*F m S*' option enabling soft filtering of low complexity regions. This option will result in the highest number of identified orthologs and minimal error rates for BLAST based identification methods [69]. In order to increase the sensitivity of the ortholog identification we applied an iterative, triangular approach searching from a given gene to all identified orthologs in other species and used them as the starting point for another search until no new ortholog were identified. This should further increase the sensitivity of the InParanoid algorithm, which has been reported to be about 80% [70], with both specificity and sensitivity, and therefore the best performing ortholog detection method [70,71]. Moreover, the test dataset used in [70] comprised six different eukaryotes (*Arabidopsis thalia, C. elegans, D. melanogaster, Homo sapiens, S. cerevisiae and S. pombe*) spanning an even broader range of the eukaryotic tree of life. To further increase the sensitivity the BLAST searches were performed on protein sequences, whereas the definition of orthology is based on genes. Therefore, the resulting ortholog clusters had to be matched to genes. Following, overlapping or identical clusters, in the case of isoforms through alternative splicing, had to be resolved. In the clearest scenario a cluster consisted of more than two proteins from one species and, after mapping to the corresponding coding gene, the cluster had two identical genes. For this cluster one of the identical genes was deleted during the collapsing process. If two independent clusters consisted of several proteins and the clusters became identical after mapping, one of this clusters was deleted. In the case of overlapping clusters after mapping the clusters were merged.

As a result of the iterative search and the possibility of false positive assignments, the specificity might decrease. As our focus was on the secondary losses and the resulting evolutionary flexibility, this should only weakly influence our predictions. Moreover, this iterative search procedure should reduce the effect of fast evolving genomes and differences in the evolutionary rate of the examined species because the ortholog prediction is not merely based on direct ortholog identification starting from human, but predicting orthologs from more closely related species.

We defined gene emergence as the point in the lineage leading to the most recent common ancestor of the species in which the ortholog genes were present [72] (see Fig. 1c). This maximum parsimony approach will give a too recent origin of the gene if it was lost in the sister group of the derived last common ancestor. Considering the species sampling, this effect might be prominent for genes lost in fungi, which will be classified as metazoan specific. Similarly, a secondary loss was defined as the point in the phylogenetic tree where no ortholog of a given gene could be identified. This could be in a species or in the last common ancestor of several species if subsequent to the ancestor no ortholog was identified [19]. Thus, no multiple independent losses were counted (see Fig. 1c).

### Interaction data

The protein-complex dataset was based on HPRD [9] version 7 (9. Jan. 2007). We extracted only data derived by affinity purification techniques leading to 1,060 complexes with 6,136 annotated proteins. The latter were mapped to 4,939 genes in total. These represented 2,197 unique genes due to homo-dimerisation of the gene products within a complex as well as gene products present in more than one complex.

### Comparison of phylogenetic pattern with random sets

To test, whether the observed evolutionary trends reflected a specific feature of protein complexes, we compared our results with a random model. We randomly drew 2,197 human genes out of the human dataset (approximately 23,000 genes). Based on this dataset, we applied the iterative ortholog detection method and retrieved the phylogenetic pattern of emergence. Moreover, based on the random dataset of 2,197 distinct genes we calculated random complexes with the same size distribution observed in the HPRD dataset (1,060 random complexes with 4,939 genes; genes must not be present twice or more in a given, but can be present in multiple complexes). We computed 10,000 repeats and compared this random model to the phylogenetic pattern observed for the HPRD dataset. As secondary losses depend on the point of emergence, we created a subset of randomly chosen 2,197 distinct genes out of the human dataset according to the observed distribution of emergence events along the tree. Furthermore, we created random complexes with the same size distribution observed in the HPRD dataset. For these dataset we computed 1,000 repeats and compared the phylogenetic pattern of secondary losses with the HPRD dataset. To estimate whether the biological signal deviated from the random model, we counted how many times a larger or lower signal, depending on the under- or overrepresentation of evolutionary events, was found in the random set. This count was divided by the number of random experiments to obtain a p-value estimate for every node. We corrected the alpha-value 0.05 for multiple testing according to the rough false discovery rate and marked the nodes with a p-value smaller than the corrected alpha as significant.

## Authors' contributions

JS designed the study. The analysis was performed by MFS. Both drafted and contributed to writing the paper, read the final manuscript and approved it.

## Supplementary Material

Additional file 1 **Gene identifier of the ortholog genes predicted for the APC complex**. Tabular collection of the obtained ortholog gene identifier of the human APC complex predicted by the iterative orthologs identification procedure and manual curation. Click here for file

Additional file 2 **Phylogenetic tree with gene and complex emergence and losses (according to the coelomata hypothesis)**. The pattern of gene and complex emergence and the secondary losses of components of whole complexes is displayed along the tree according to the absence and presence pattern of the ortholog genes in terminal species or in subsets of species concluding the loss in the last common ancestor of all subsequent species. The numbers of gene and complex emergence is indicated in blue (complex emergence/gene emergence). The number of secondary losses are shown in red per affected node. It was discriminated between whole complex losses and gene losses (complex loss/gene losses). Click here for file
