# Supplementary material for: Evolutionary flexibility of protein complexes
Source: BMC Evol Biol. 2009 Jul 7;9:155. doi: 10.1186/1471-2148-9-155 (PMC3224664; doi:10.1186/1471-2148-9-155)
Supplement: Additional file 1 — Gene identifier of the ortholog genes predicted for the APC complex. Tabular collection of the obtained ortholog gene identifier of the human APC complex predicted by the iterative orthologs identification procedure and manual curation. [file 1471-2148-9-155-S1.pdf]

	APC1	APC4	APC5	APC2	APC11	APC10	APC8	APC6	APC3	APC7	CDC26
Homo_sapiens	ENSG00000153107	ENSG00000053900	ENSG00000089053	ENSG00000176248	ENSG00000141552	ENSG00000164162	ENSG00000094880	ENSG00000130177	ENSG00000004897	ENSG00000196510	ENSG00000176386
Mus_musculus	ENSMUSG00000014355	ENSMUSG00000029176	ENSMUSG00000029472	ENSMUSG00000026965	ENSMUSG00000025135	ENSMUSG00000036977	ENSMUSG00000024370	ENSMUSG00000038416	ENSMUSG00000020687	ENSMUSG00000029466	ENSMUSG00000058745
Rattus_norvegicus	ENSRNOG00000016965	ENSRNOG00000004130	ENSRNOG00000001316	ENSRNOG00000011295	ENSRNOG00000036686	ENSRNOG00000018296	ENSRNOG00000024241	ENSRNOG00000017536	ENSRNOG00000005904	ENSRNOG00000001283	ENSRNOG00000029785
Xenopus_tropicalis	ENSXETG00000000782	ENSXETG00000000957	ENSXETG00000020963	ENSXETG00000022932	496610	ENSXETG00000017613	ENSXETG00000006716	ENSXETG00000002573	ENSXETG00000003238	ENSXETG00000020941	-
Oryzias_latipes	ENSORLG00000003899	ENSORLG00000000318	ENSORLG00000014538	ENSORLG00000001114	ENSORLG00000001673	ENSORLG00000002856	ENSORLG00000006778	ENSORLG00000010961	ENSORLG00000019533	ENSORLG00000015911	ENSORLG00000019646
Tetraodon_nigroviridis	GSTENG00014297001	GSTENG00032787001	GSTENG00021646001	GSTENG00021292001	GSTENG00026387001	GSTENG00008031001	GSTENG00019048001	GSTENG00027898001	GSTENG00005521001	GSTENG00021598001	GSTENG00024792001
Takifugu_rubripes	SINFRUG00000162817	SINFRUG00000163090	SINFRUG00000121640	SINFRUG00000125021	SINFRUG00000125752	SINFRUG00000151657	SINFRUG00000149925	SINFRUG00000131623	SINFRUG00000150982	SINFRUG00000134919	-
Danio_rerio	568795	ENSDARG00000032237	ENSDARG00000008461	ENSDARG00000035832	ENSDARG00000070333	ENSDARG00000060816	ENSDARG00000044484	ENSDARG00000055470	ENSDARG00000056258	ENSDARG00000063005	ENSDARG00000067640
Ciona_intestinalis	ENSCING00000005956	ENSCING00000017492	ENSCING00000014310	ENSCING00000002661	100185352	ENSCING00000009153	ENSCING00000006939	ENSCING00000008016	ENSCING00000007478	ENSCING00000004134	-
Branchiostoma_floridae	fgenesh2_pg.scaffold_231000046	estExt_fgenesh2_pg.C_1350038	fgenesh2_pg.scaffold_351000043	estExt_fgenesh2_pg.C_110185	fgenesh2_pg.scaffold_216000060	estExt_fgenesh2_pg.C_240116	estExt_fgenesh2_pg.C_1490046	estExt_fgenesh2_pg.C_810036	estExt_fgenesh2_pg.C_120181	estExt_GenewiseH_1.C_6070018	estExt_fgenesh2_pg.C_1930034
Apis_mellifera	GB19084	GB18982	GB18016	GB14760	GB17329	GB16034	GB19210	GB18057	GB16427	GB13555	GB30521
Anopheles_gambiae	AGAP000374	AGAP002345	AGAP005578	AGAP005048	AGAP008196	AGAP011799	AGAP009880	AGAP004623	AGAP007690	AGAP003902	-
Drosophila_melanogaster	CG9198	CG32707	CG10850	CG3060	CG34440	CG11419	CG2508:CG31687	CG6759	CG8610	CG14444	-
Daphnia_pulex	e_gw1.1.406.1	e_gw1.39.115.1	SNAP_00030114	NCBI_GNO_0400531	PASA_GEN_6000031	PASA_GEN_10700012	e_gw1.8.64.1	PASA_GEN_0300213	PASA_GEN_0100383	PASA_GEN_21100021	-
Caenorhabditis_elegans	W10C6.1	F54C8.3	176583	K06H7.6	175604	Y48G1C.12	F10C5.1	F10B5.6	Y110A7A.17	-	-
Nematostella_vectensis	fgenesh1_pg.scaffold_89000037	e_gw.299.68.1	estExt_fgenesh1_pg.C_2280020	e_gw.287.30.1	estExt_fgenesh1_pm.C_1700003	estExt_fgenesh1_pg.C_1180053	fgenesh1_pg.scaffold_50000019	estExt_GenewiseH_1.C_300075	gw.44.85.1	e_gw.75.12.1	-
Trichoplax_adhaerens	fgeneshTA2_pg.C_scaffold_1001403	fgeneshTA2_pg.C_scaffold_4000262	fgeneshTA2_pg.C_scaffold_2000544	gw1.9.50.1	e_gw1.12.177.1	e_gw1.2.1518.1	fgeneshTA2_pg.C_scaffold_2000395	e_gw1.11.317.1	gw1.3.1317.1	fgeneshTA2_pg.C_scaffold_1000352	-
Monosiga_brevicollis	fgenesh2_pg.scaffold_2000357	fgenesh2_pg.scaffold_5000337	-	fgenesh2_pg.scaffold_28000014	-	fgenesh2_pg.scaffold_13000157	fgenesh2_pg.scaffold_16000058	estExt_fgenesh2_pg.C_100067	fgenesh2_pg.scaffold_16000068	estExt_fgenesh2_pg.C_60324	-
Schizosaccharomyces_pombe	SPBC106.09	SPAC19G12.01c	SPAC959.09c	SPBP23A10.04	SPAC343.03	SPBC1A4.01	SPAC6F12.14	SPAC6F12.15c	SPAC17C9.01c	-	-
Saccharomyces_cerevisiae	YNL172W	-	YOR249C	YLR127C	YDL008W	YGL240W	YHR166C	YKL022C	YBL084C	-	-
Aspergillus_niger	estExt_fgenesh1_pg.C_40821	fgenesh1_pg.C_scaffold_1000086	estExt_fgenesh1_pg.C_40783	gw1.4.821.1	gw1.12.314.1	fgenesh1_pg.C_scaffold_1000081	fgenesh1_pg.C_scaffold_2000747	e_gw1.2.1252.1	fgenesh1_pg.C_scaffold_9000435	-	-
Laccaria_bicolor	gww1.9.149.1	estExt_fgenesh2_pg.C_10083	eu2.Lbscf0002g10120	fgenesh3_pg.C_scaffold_18000148	eu2.Lbscf0057g00660	eu2.Lbscf0011g04980	eu2.Lbscf0073g00560	gww1.11.377.1	eu2.Lbscf0006g06770	-	-
Phycomyces_blakesleeanus	estExt_fgeneshPB_pg.C_10665	fgeneshPB_pg.3__208	fgeneshPB_pg.43__50	fgeneshPB_pg.9__55	e_gw1.2.943.1	fgeneshPB_pg.23__223	fgeneshPB_pg.7__417	estExt_fgeneshPB_pg.C_50273	fgeneshPB_pg.6__305	-	-
Encephalitozoon_cuniculi	-	-	-	-	-	859107	-	-	-	-	-
