# Supplementary figures and images for: Evolutionary flexibility of protein complexes
Source: BMC Evol Biol. 2009 Jul 7;9:155. doi: 10.1186/1471-2148-9-155 (PMC3224664; doi:10.1186/1471-2148-9-155)

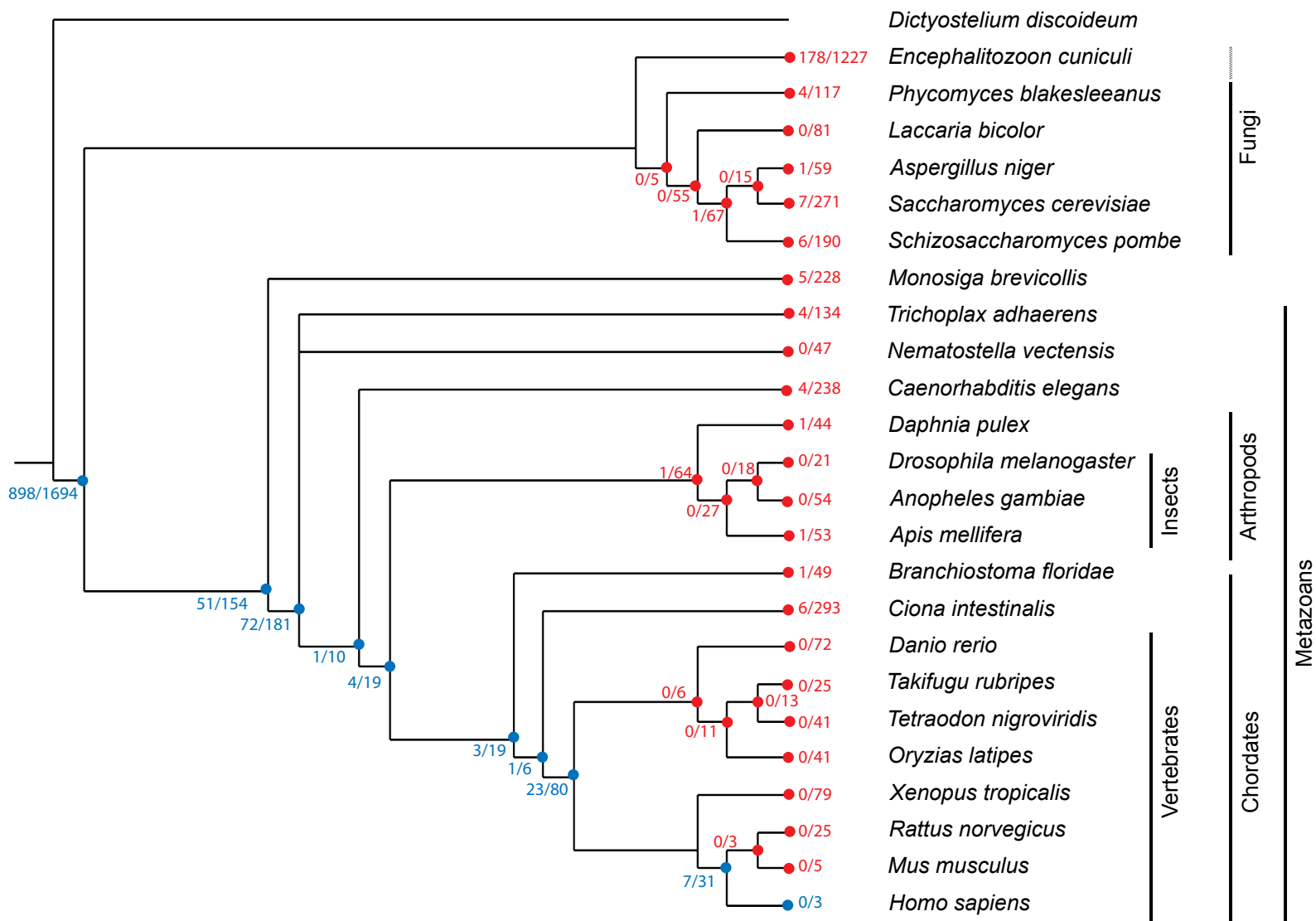

Supplement: Additional file 2 — Phylogenetic tree with gene and complex emergence and losses (according to the coelomata hypothesis). The pattern of gene and complex emergence and the secondary losses of components of whole complexes is displayed along the tree according to the absence and presence pattern of the ortholog genes in terminal species or in subsets of species concluding the loss in the last common ancestor of all subsequent species. The numbers of gene and complex emergence is indicated in blue (complex emergence/gene emergence). The number of secondary losses are shown in red per affected node. It was discriminated between whole complex losses and gene losses (complex loss/gene losses). [file 1471-2148-9-155-S2.pdf]
